# MetaboSignal: a network-based approach for topological analysis of metabotype regulation *via* metabolic and signaling pathways

**DOI:** 10.1093/bioinformatics/btw697

**Published:** 2016-12-05

**Authors:** Andrea Rodriguez-Martinez, Rafael Ayala, Joram M Posma, Ana L Neves, Dominique Gauguier, Jeremy K Nicholson, Marc-Emmanuel Dumas

**Affiliations:** 1 Computational and Systems Medicine, Department of Surgery and Cancer, Faculty of Medicine, Imperial College London, London, UK; 2 Sorbonne Universities, University Pierre & Marie Curie, University Paris Descartes, Sorbonne Paris Cité, INSERMUMR_S, Cordeliers Research Centre, Paris, France

## Abstract

**Summary:**

MetaboSignal is an R package that allows merging metabolic and signaling pathways reported in the Kyoto Encyclopaedia of Genes and Genomes (KEGG). It is a network-based approach designed to navigate through topological relationships between genes (signaling- or metabolic-genes) and metabolites, representing a powerful tool to investigate the genetic landscape of metabolic phenotypes.

**Availability and Implementation:**

MetaboSignal is available from Bioconductor: https://bioconductor.org/packages/MetaboSignal/

**Supplementary information:**

Supplementary data are available at *Bioinformatics* online.

## 1 Introduction

‘Omics’ approaches such as genomics, metabolomics and metabonomics are used to improve our understanding of integrated functioning of living organisms, with each ‘omics’ generating high-density datasets (Dumas, 2012; Nicholson *et al.*, 2002). Genes and metabolites provide complementary information about biological processes. Metabolic reactions are catalyzed by enzymes encoded by genes. The activity of these enzymes is regulated by transcriptional, translational and post-translational mechanisms impacting metabolism. Metabolic processes are also regulated by signaling transduction pathways, allowing the organism to adapt to environmental changes and maintain homeostasis.

The Kyoto Encyclopaedia of Genes and Genomes (KEGG) (Kanehisa and Goto, 2000) is a popular reference database storing biological pathways and cellular processes from a wide range of reference organisms as graphical diagrams, where the nodes are biological entities (e.g. genes, metabolites) and the edges represent the relationship between them. In the recent years, several tools have been developed for analyzing, editing and customizing KEGG metabolic (Cottret *et al.*, 2010; Posma *et al.*, 2014) or signaling (Zhang and Wiemann, 2009) pathways independently. However, these tools ignore the interconnection between the genome and metabolome, and therefore they do not provide an integrated overview of regulatory events leading to the observed metabolic patterns.

We introduced an integrated metabolome and interactome mapping (iMIM) strategy to analyze the topology of protein interaction, signaling and metabolic networks, and implemented it as a standalone database and workflow (Davidovic *et al.*, 2011). To make this network-modeling strategy accessible to a wider community, we have now developed an R-based approach, called MetaboSignal, which allows building organism-specific KEGG networks that account for the interaction between metabolites and genes (both metabolic and signaling). Advantages of MetaboSignal over other network-based approaches include: (i) network directionality; (ii) network filtering based on KEGG pathway and tissue-specific gene expression; (iii) optional clustering of genes into ortholog groups, which enables comparing networks from different organisms; (iv) topological exploration of gene-metabolite associations based on network statistics.

## 2 Methods and features

First, relevant metabolic and signaling pathways are parsed using KEGGgraph (Zhang and Wiemann, 2009). Parsed pathways are then used to build organism-specific metabolic and signaling directed network-tables (i.e. 2-column matrix). The metabolic network is formalized as a tripartite network with the following types of nodes: metabolites, metabolic-genes involved in enzymatic reactions, and non-enzymatic (e.g. spontaneous) reactions. Likewise, the signaling network is a tripartite network with: signaling-genes (e.g. kinases), metabolic-genes and metabolites. The user can decide whether gene nodes represent organism-specific gene IDs or orthology IDs. MetaboSignal also gives the option to build a tissue-specific signaling network that excludes genes not expressed in a given tissue. Tissue-specific pruning is achieved using information from the Human Protein Atlas database (Gatto, 2016; Uhlen *et al.*, 2010).

The metabolic network is then merged with the signaling network to create the ‘MetaboSignal’ network that can be customized according to different criteria. For instance, undesired nodes can be removed or nodes representing isomers of the same molecule (e.g. α and β d-glucose) can be collapsed into a single node. MetaboSignal analyzes network topology using the igraph R-package (Csardi, 2015) to: (i) derive a gene-metabolite distance matrix based on shortest path lengths, (ii) compute centrality statistics and (iii) generate concise sub-networks based on betweenness-ranked shortest paths from a list of input genes to a list of metabolites.

Finally, the original network or the sub-networks can be visualized in R. Alternatively, MetaboSignal allows generating a network file and several attribute files to visualize and customize the network in Cytoscape (Shannon *et al.*, 2003). For further details, see supplementary information.

## 3 Example

To illustrate the functionality of our approach, we used transcriptomic and metabonomic datasets from the adipose tissue of rat congenic strains derived from the diabetic Goto-Kakizaki (GK) and normoglycemic Brown-Norway (BN) rats (data and accession numbers available from Dumas *et al.*, 2016). Quantitative-trait locus (QTL) analysis of these datasets identified eQTL- and mQTL-responsive genes and metabolites.

**Fig. 1.**
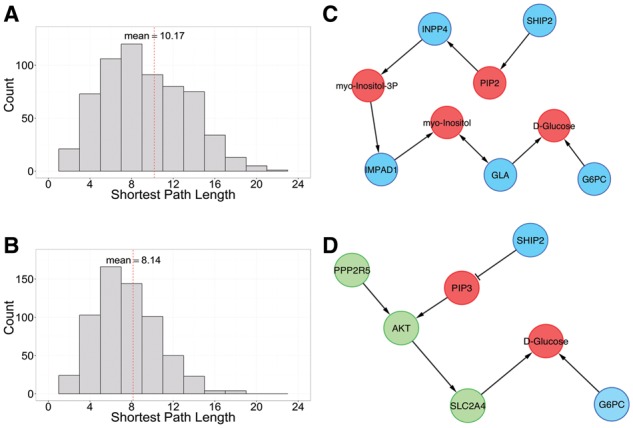
Example of interconnection between the genome and metabolome using a rat adipose-tissue dataset. (**A**, **B**) Histograms of shortest path lengths from 38 metabolic-genes to 19 metabolites in (A) the metabolic network and in (B) the MetaboSignal network. (**C**, **D**) Comparison of shortest paths from *G6pc3* (G6PC) or *Ship2* (SHIP2) to D-glucose in (C) the metabolic network and in (D) the MetaboSignal network. Panel D also shows the shortest path from *Ppp2r5b* (PPP2R5) to d-glucose. Node color represents: metabolic-genes (blue), signaling-genes (green) and metabolites (red)

A MetaboSignal network was built using all metabolic and signaling KEGG pathways from the rat adipose-tissue. 19 metabolites and 58 genes (38 metabolic- and 20 signaling-genes) significantly associated with one locus were mapped onto the MetaboSignal network. We computed shortest path lengths from the 38 metabolic-genes to the 19 metabolites in both the metabolic network (without signaling-genes, Fig. 1A) and MetaboSignal network (Fig. 1B). 52% of the reachable paths were shorter in the MetaboSignal network reflecting the complementarity between metabolic and signaling transduction pathways.

Finally, to illustrate the interconnectivity between metabolites, metabolic-genes and signaling-genes, we built sub-networks containing the shortest paths from genes to metabolites with significant differential abundance in the same genomic region. Figure 1C and D compares the shortest path from *G6pc3* or *Ship2* to d-glucose in the metabolic network (C) or in the MetaboSignal network (D). For *G6pc3*, the shortest path is equivalent in both networks, since d-glucose is the product of the reactions catalyzed by this gene (‘rn:R00303’, ‘rn:R01788’). In the case of *Ship2*, the MetaboSignal network allows shortening the distance to D-glucose from 7 to 4 steps and provides a more biologically relevant explanation for this gene-metabolite association. Thus, SHIP2 dephosphorylates phosphatidylinositol 3,4,5-trisphosphate (PIP3), a second messenger playing a key role in the activation of AKT (Wada *et al.*, 2001). AKT regulates D-glucose uptake in adipocytes *via* SLC2A4 (glucose transporter 4). Figure 1D also shows the shortest path between D-glucose and *Ppp2r5b*, which directly inhibits AKT *via* dephosphorylation (Rodgers *et al.*, 2011).

## 4 Discussion

‘MetaboSignal’ is a versatile package integrating metabolic and signaling transduction pathways to build parsimonious visualizations of gene-metabolite associations based on the analysis of network topology. MetaboSignal is a biomolecular navigation system allowing the exploration of organism-specific, and even tissue-specific, relationships between any given gene and any given metabolite retrieved from KEGG. This approach is ideally suited to identify candidate genes in metabotype-QTL studies (e.g. *trans-*acting associations), or to identify biological pathways affected in transgenic models (e.g. knock-out, CRISPR-Cas9) (Dumas, 2012). Finally, MetaboSignal is easily amenable to incorporate other pathway databases and types of interactions, such as protein interactions and transcription factor networks.

## Funding

Medical Research Council Doctoral Training Centre PhD scholarship (MR/K501281/1), Imperial College PhD-scholarship (EP/M506345/1), *La Caixa* studentship to ARM. Portuguese Foundation for Science and Technology (SFRH/BD/52036/2012) to ALN. Grants from the European Commission (FGENTCARD, LSHG-CT-2006-037683, EURATRANS, HEALTH-F4-2010-241504, METACARDIS, HEALTH-F4-2012-305312) to DG, JKN and MED.

*Conflict of Interest*: none declared.

## Supplementary Material

Supplementary Data

## References

[btw697-B1] Csardi G. (2015) igraph package. The Comprehensive R Archive Network, v1.0.1,

[btw697-B2] Cottret L. et al (2010) Metexplore: a web server to link metabolomic experiments and genome-scale metabolic networks. Nucleic Acids Res., 38, W132–W137. 20444866 10.1093/nar/gkq312 PMC2896158

[btw697-B3] Davidovic L. et al (2011) A metabolomic and systems biology perspective on the brain of the fragile X syndrome mouse model. Genome Res., 21, 2190–2202. 21900387 10.1101/gr.116764.110 PMC3227107

[btw697-B4] Dumas M.E. (2012) Metabolome 2.0: quantitative genetics and network biology of metabolic phenotypes. Mol. Biosyst., 8, 2494–2502. 22868675 10.1039/c2mb25167a

[btw697-B5] Dumas M.E. et al (2016) Topological analysis of metabolic networks integrating co-segregating transcriptomes and metabolomes in type 2 diabetic rat congenic series. Genome Med., 8, 101. 27716393 10.1186/s13073-016-0352-6 PMC5045612

[btw697-B6] Gatto L. (2016) hpar: Human Protein Atlas in R. Bioconductor, v1.14.2,

[btw697-B7] Kanehisa M., Goto S. (2000) KEGG: Kyoto Encyclopedia of Genes and Genomes. Nucleic Acids Res., 28, 27–30. 10592173 10.1093/nar/28.1.27 PMC102409

[btw697-B8] Nicholson J.K. et al (2002) Metabonomics: a platform for studying drug toxicity and gene function. Nat. Rev. Drug Discov., 1, 153–161. 12120097 10.1038/nrd728

[btw697-B9] Posma J.M. et al (2014) MetaboNetworks, an interactive Matlab-based toolbox for creating, customizing and exploring sub-networks from KEGG. Bioinformatics, 30, 893–895. 24177720 10.1093/bioinformatics/btt612 PMC3957072

[btw697-B10] Rodgers J.T. et al (2011) Clk2 and B56β mediate insulin-regulated assembly of the PP2A phosphatase holoenzyme complex on Akt. Mol. Cell, 41, 471–479. 21329884 10.1016/j.molcel.2011.02.007 PMC3060660

[btw697-B11] Shannon P. et al (2003) Cytoscape: a software environment for integrated models of biomolecular interaction networks. Genome Res., 13, 2498–2504. 14597658 10.1101/gr.1239303 PMC403769

[btw697-B12] Uhlen M. et al (2010) Towards a knowledge-based Human Protein Atlas. Nat. Biotechnol., 28, 1248–1250. 21139605 10.1038/nbt1210-1248

[btw697-B13] Wada T. et al (2001) Overexpression of SH2-containing inositol phosphatase 2 results in negative regulation of insulin-induced metabolic actions in 3T3-L1 adipocytes via its 5′-phosphatase catalytic activity. Mol. Cell Biol., 21, 1633–1646. 11238900 10.1128/MCB.21.5.1633-1646.2001 PMC86709

[btw697-B14] Zhang J.D., Wiemann S. (2009) KEGGgraph: a graph approach to KEGG PATHWAY in R and bioconductor. Bioinformatics, 25, 1470–1471. 19307239 10.1093/bioinformatics/btp167 PMC2682514

