# Supplementary material for: MetaboSignal: a network-based approach for topological analysis of metabotype regulation via metabolic and signaling pathways
Source: Bioinformatics. 2016 Dec 5;33(5):773–5. doi: 10.1093/bioinformatics/btw697 (PMC5408820; doi:10.1093/bioinformatics/btw697)

MetaboSignal, a network-based approach to overlay metabolic and signaling pathways to study metabolic phenotypes

Andrea Rodriguez-Martinez[1], Rafael Ayala[1], Joram M. Posma[1], Ana L. Neves[1], Dominique Gauguier[1,2], Jeremy K. Nicholson[1] and Marc-Emmanuel Dumas[1*]

[1]Computational and Systems Medicine, Department of Surgery and Cancer, Faculty of Medicine, Imperial College London, SW7 2AZ, London, United Kingdom.

[2]Sorbonne Universities, University Pierre & Marie Curie, University Paris Descartes, Sorbonne Paris Cité, INSERMUMR_S 1138, Cordeliers Research Centre, 75006 Paris, France.

Supplementary material

In this document, we provide the following information:

- High-resolution version of the figure shown in the main paper.
- Glossary and reference information for MetaboSignal.
- Instructions for how to install and get started with MetaboSignal.
- Description of all the functionalities of MetaboSignal, with illustrative examples.
- Common workflow for how MetaboSignal can be used.
- Basic guidelines about how to import, visualize and customize MetaboSignal networks in cytoscape.

Main paper figure

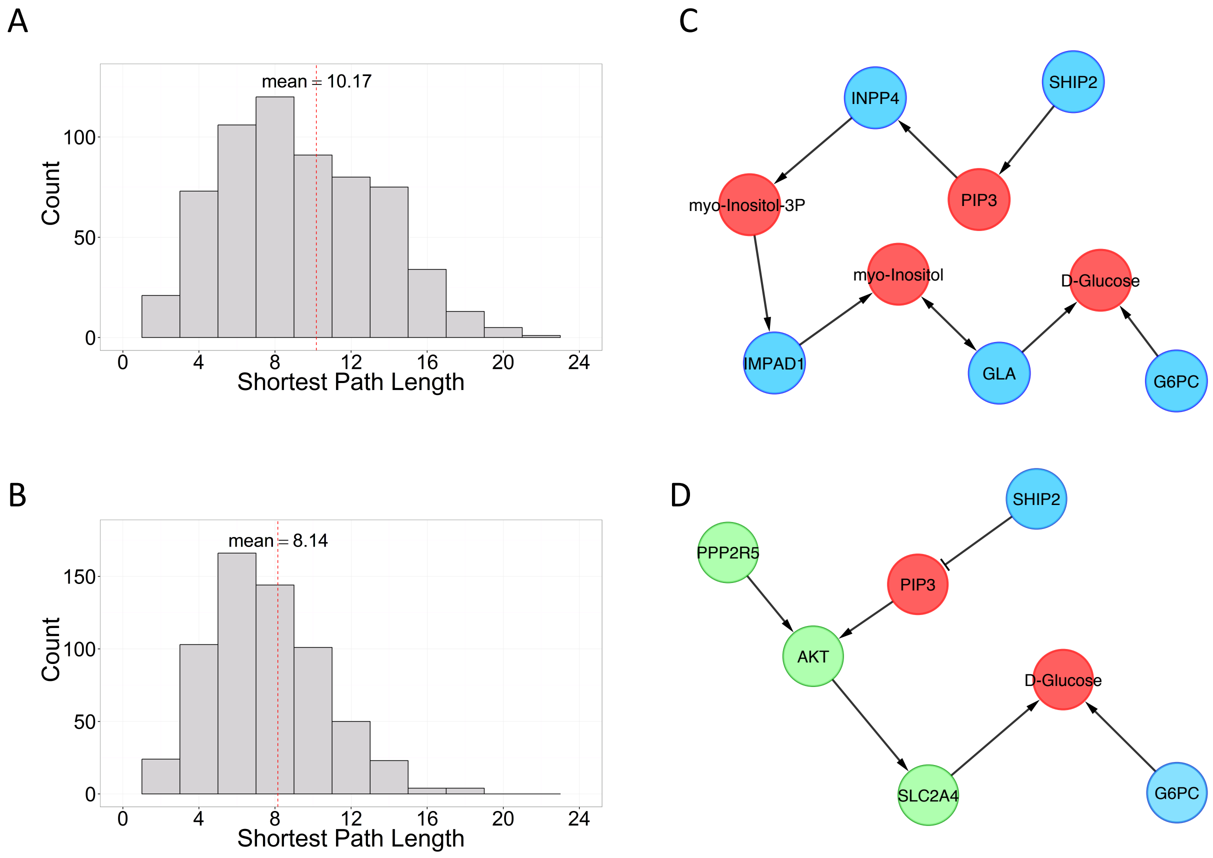

Fig. 1. Example of interconnection between the genome and metabolome using a rat adipose-tissue dataset. (A, B) Histograms of shortest path lengths from 38 metabolic-genes to 19 metabolites in (A) the metabolic network and in (B) the MetaboSignal network. (C, D) Comparison of shortest paths from *G6pc3* (G6PC) or *Ship2* (SHIP2) to D-glucose in (C) the metabolic network and in (D) the MetaboSignal network. Panel D also shows the shortest path from *Ppp2r5b* (PPP2R5) to D-glucose. Node color represents: metabolic-genes (blue), signaling-genes (green) and metabolites (red).

Glossary terms and reference information

KEGG pathway: collection of pathway maps representing reaction and molecular interaction networks (http://www.genome.jp/kegg/pathway.html).

Metabolic pathway: KEGG pathway map included in the category “Metabolism”.

Signaling pathway: KEGG pathway map included in one of the following categories: “Environmental Information Processing”, “Cellular Processes”, “Organism Systems”, “Human Diseases”.

KEGG Orthology (KO) database: KEGG database where molecular-level functions are associated in ortholog groups (http://www.genome.jp/kegg/ko.html). Clustering genes into orthology groups enables comparing networks from different organisms. It also allows reducing network dimensionality without loosing functional information.

MetaboSignal network: network generated by merging metabolic and signaling KEGG pathways. This network is generated using the function “MetaboSignal_matrix( )”, which involves the main following steps:

- First, relevant metabolic and signaling KEGG pathways are parsed using the KEGGgraph package (http://bioconductor.org/packages/release/bioc/html/KEGGgraph.html).
- Parsed pathways are then used to build directed metabolic and signaling networks formalized as 2-column matrices, each row of the matrix representing an edge between two nodes.
- The original metabolic network has two types of nodes: reactions and metabolites. Reactions are linked to enzymes, and then to genes using the KEGG API (http://www.genome.jp/kegg/rest/keggapi.html).
- Optionally, the genes from the signaling network can be filtered based on tissue-expression using information from the Human Protein Atlas database (http://www.proteinatlas.org/).
- Finally, the metabolic and signaling networks are merged (i.e. “rbind()” of the 2 matrices) to build the MetaboSignal network.

Node types: MetaboSignal networks are composed by 4 different types of nodes:

- Metabolic genes: genes encoding enzymes that catalyze metabolic reactions.
- Signaling genes: genes encoding regulatory proteins involved in signaling pathways (e.g. kinases, phosphatases, receptors).
- Metabolites (http://rest.kegg.jp/list/compound).
- Non-enzymatic reactions: spontaneous reactions that are not catalyzed by enzymes (e.g. “rn:R07420” involving the cyclization of creatine into creatinine) or reactions that cannot be linked to enzymes in the KEGG API (http://rest.kegg.jp/link/rn/ec) (e.g. “rn:R10507”).

Node betweenness: measurement of node centrality defined as the number of shortest paths going through a given node of the network (http://igraph.org/c/doc/igraph-docs.pdf).

Shortest path: path connecting two nodes with the least number of edges. Shortest paths are calculated using the breadth-first algorithm (http://igraph.org/c/doc/igraph-docs.pdf).

Installation instructions

To use MetaboSignal, R **(version >= 3.3)** must be correctly installed. Here, we strongly recommend using RStudio.

MetaboSignal is currently available from the devel version of Bioconductor. For details, check: https://bioconductor.org/packages/MetaboSignal/

To install MetaboSignal, start R (or RStudio) and follow the instructions shown below:

```
# 1) Install BiocInstaller and BiocGenerics
source("https://bioconductor.org/biocLite.R")
biocLite(c("BiocInstaller", "BiocGenerics"), ask = FALSE)

# 2) Install MetaboSignal
source("https://bioconductor.org/biocLite.R")
BiocInstaller::useDevel()
biocLite("MetaboSignal", ask=FALSE)
```

Important note: MetaboSignal depends on **hpar version > = 1.15.3.**

If prompted with the message: “Do you want to install from sources packages which need compilation?”, choose no.

3) Remove package

To remove the package use: remove.packages("MetaboSignal") and restart R.

Get started

Under R, and after having installed MetaboSignal, load the package using:

```
library(MetaboSignal)
```

To see the vignette of the package use:

```
browseVignettes("MetaboSignal")
```

To get help with any function (e.g. MetaboSignal_matrix) use:

```
help("MetaboSignal_matrix")
```

MetaboSignal core functions

MetaboSignal_matrix

Description

This function generates a directed network-table (i.e. two-column matrix), where each row represents an edge connecting two nodes (from node in column 1 to node in column 2). Nodes represent four different molecular entities: metabolic-genes (i.e. genes encoding enzymes that catalyze metabolic reactions), signaling-genes (e.g. kinases), reactions or metabolites. It is possible to build a tissue-specific network-table that excludes signaling genes that are not expressed in a given tissue. Tissue expression data is obtained using the hpar package, which is based on the The Human Protein Atlas database. The genes "non detected" in the target tissue (reliability = supportive) are neglected.

The network-table generated with this function can be customized based on several criteria. For instance, undesired nodes can be removed or replaced using the functions "MS_RemoveNode( )" or "MS_ReplaceNode( )" respectively. Also, the network can be filtered according to different topological parameters (e.g. node betweenness) using the function "MS_FilterNetwork( )".

Usage

```
MetaboSignal_matrix(metabo_paths, signaling_paths, organism_name, tissue = "all",
    expand_genes = FALSE)
```

Arguments

metabo_paths: character vector containing the KEGG IDs of the metabolic pathways of interest (organism-specific). For example, the KEGG ID for the pathway "glycolysis/gluconeogenesis" in the rat is "rno00010". See the function "MS_FindKEGG( )".

signaling_paths: character vector containing the KEGG IDs for the signaling pathways of interest (organism-specific). For example, the KEGG ID for the pathway "insulin signaling pathway" in the rat is "rno04910".

organism_name: character vector containing the common name of the organism of interest (e.g. "rat", "mouse", "human", "zebrafish") or taxonomy id. For more details, check: http://docs.mygene.info/en/latest/doc/data.html#species. This argument is only required when filtering genes by tissue expression.

tissue: character vector containing the name(s) of the target tissue(s). By default, tissue = "all" indicating that signaling gene nodes will not be filtered by tissue expression. Otherwise, possible tissues are those included in the dataset hpaNormalTissue (see levels(hpaNormalTissue$Tissue).

expand_genes: logical scalar indicating whether the gene nodes will represent orthology IDs (FALSE) or organim-specific gene IDs (TRUE). By default, expand_genes = FALSE.

Value
A two-column matrix where each row represents an edge between two nodes.

Note

Reaction directionality reported in KEGG has been cross-validated with published literature. For more details, check the dataset directionality_reactions.

Examples
```
# MetaboSignal_table with organism-specific gene nodes
MetaboSignal_tableIsoforms <- MetaboSignal_matrix(metabo_paths = c("rno00010",
   "rno00562"), signaling_paths = c("rno04910", "rno04151"), expand_genes = TRUE)

# MetaboSignal_table with orthology gene nodes
MetaboSignal_table <- MetaboSignal_matrix(metabo_paths = c("rno00010",
   "rno00562"), signaling_paths = c("rno04910", "rno04151"))

# MetaboSignal_table with orthology gene nodes filtered by liver
MetaboSignal_tableLiver <- MetaboSignal_matrix(metabo_paths = "hsa00010",
   signaling_paths = "hsa04151", organism_name = "human",  tissue = "liver")
```

MetaboSignal_distances

Description
This function generates a distance matrix containing the length of all shortest paths from a set of genes (or reactions) to a set of metabolites. The shortest path length between two nodes is defined as the minimum number of edges between these two nodes.

Usage
```
MetaboSignal_distances(network_table, organism_code, organism_name, mode = "SP",
   source_genes = "all", target_metabolites = "all", names = FALSE)
```

Arguments
network_table: two-column matrix where each row represents an edge between two nodes. See function "MetaboSignal_matrix ( )".

organism_code: character vector containing the KEGG code for the organism of interest. For example the KEGG code for the rat is "rno". See the function "MS_FindKEGG( )".

organism_name: character vector containing the common name of the organism of interest (e.g. "rat", "mouse", "human", "zebrafish") or taxonomy id. For more details, check: http://docs.mygene.info/en/latest/doc/data.html#species. This argument is only required when source_genes are gene symbols.

mode: character constant indicating whether a directed or an undirected network will be considered. "all" indicates that all the edges of the network will be considered as undirected.

"out" indicates that all the edges of the network will be considered as directed. "SP" indicates that all network will be considered as directed except the edges linked to target metabolite, which will be considered as undirected. The difference between the "out" and the "SP" options is that the latter aids reaching target metabolites that are substrates of irreversible reactions. By default, mode = "SP".

source_genes: character vector containing the genes from which the shortest paths will be calculated. All input genes need to have the same ID format. Possible ID formats are: entrez IDs, official gene symbols, or gene nodes of the network (i.e. KEGG orthology IDs or KEGG gene IDs). The latter option allows reducing the time required to compute this function. Entrez IDs or gene symbols can be transformed into KEGG IDs using the function "MS_GetKEGG_GeneID( )". By default, genes = "all" indicating that all genes or reactions of the network will be used.

target_metabolites: character vector containing the KEGG IDs of the metabolites to which the shortest paths will be calculated. Compound KEGG IDs can be obtained using the function "MS_FindKEGG( )". By default, metabolites = "all", indicating that all metabolites of the network will be used.

names: logical scalar indicating whether the metabolite IDs or gene KEGG IDs will be transformed into common metabolite names or gene symbols. Reaction IDs remain unchanged. By default, names = FALSE.

Value

A matrix containing the shortest path length from the genes or reactions (in the rows) to the metabolites (in the columns). For unreacheable metabolites Inf is included.

Examples

Distances from all genes to all metabolites of the network

```
distances_all <- MetaboSignal_distances(MetaboSignal_table, organism_code = "rno")
```

Distances from *Ship2* (65038) and *Ppp2r5b* (309179) to D-glucose ("cpd:C00031")

```
distances_targets <- MetaboSignal_distances(MetaboSignal_table, organism_code =
   "rno", source_genes = c("65038", "309179"), target_metabolites = "cpd:C00031",
      names = TRUE)
```

##### MetaboSignal_NetworkCytoscape ####

Description

This function allows calculating the shortest paths from a set of genes to a set of metabolites, and representing them as a network-table (i.e. two-column matrix). By default, the function exports a network file ("CytoscapeNetwork.txt") and two attribute files ("CytoscapeAttributesType.txt", "CytoscapeAttributesTarget.txt"), which can be imported into cytoscape to visualize the network. The first attribute file allows customizing the nodes of the network based on the molecular entity they represent: metabolic-genes, signaling-genes, or

metabolites. The second attribute file allows discriminating the source_genes and the target_metabolites ("target") from any other node ("untarget") of the network.

The network-table generated with this function can be further customized based on different criteria. For instance, undesired nodes can be removed or replaced using the functions "MS_RemoveNode( )" or "MS_ReplaceNode( )" respectively. The final version of the network-table can be used to generate new cytoscape files using the function "MS_ToCytoscape( )".

Usage

MetaboSignal_NetworkCytoscape(network_table, organism_code, organism_name,
    source_genes, target_metabolites, mode = "SP", type = "first", distance_th = Inf,
        collapse_genes = FALSE, names = TRUE, export_cytoscape = TRUE, file_name =
            "Cytoscape")

Arguments

network_table: two-column matrix where each row represents an edge between two nodes. See function "MetaboSignal_matrix ( )".

organism_code: character vector containing the KEGG code for the organism of interest. For example the KEGG code for the rat is "rno". See the function "MS_FindKEGG( )".

organism_name: character vector containing the common name of the organism of interest (e.g. "rat", "mouse", "human", "zebrafish") or taxonomy id. For more details, check: http://docs.mygene.info/en/latest/doc/data.html#species. This argument is only required when source_genes are gene symbols.

source_genes: character vector containing the genes from which the shortest paths will be calculated. All input genes need to have the same ID format. Possible ID formats are: entrez IDs, official gene symbols, or gene nodes of the network (i.e. KEGG orthology IDs or KEGG gene IDs). The latter option allows reducing the time required to compute this function. Entrez IDs or gene symbols can be transformed into KEGG IDs using the function "MS_GetKEGG_GeneID( )".

target_metabolites: character vector containing the KEGG IDs of the metabolites to which the shortest paths will be calculated. Compound KEGG IDs can be obtained using the function "MS_FindKEGG( )".

mode: character constant indicating whether a directed or an undirected network will be considered. "all" indicates that all the edges of the network will be considered as undirected. "out" indicates that all the edges of the network will be considered as directed. "SP" indicates that all network will be considered as directed except the edges linked to target metabolite, which will be considered as undirected. The difference between the "out" and the "SP" options, is that the latter aids reaching target metabolites that are substrates of irreversible reactions. By default, mode = "SP".

type: character constant indicating whether all shortest paths or a single shortest path will be considered when there are several shortest paths between a source_gene and a target_metabolite. If type = "all", all shortest paths will be considered. If type = "first" a single

path will be considered. If type = "bw" the path with the highest betweenness score will be considered. The betweenness score is calculated as the average betweenness of the gene nodes of the path. Note that using type = "bw" increases the time required to compute this function. By default, type = "first".

names: logical scalar indicating whether the metabolite IDs or gene KEGG IDs will be transformed into common metabolite names or gene symbols. Reaction IDs remain unchanged. By default, names = FALSE.

export_cytoscape: logical scalar indicating whether network and attribute cytoscape files will be generated and exported. By default, export_cytoscape = TRUE.

file_name: character vector that allows customizing the name of the exported files. By default, file_name = "Cytoscape".

Value

A two-column matrix where each row represents an edge between two nodes. By default, the function also generates a network file ("CytoscapeNetwork.txt") and two attribute files ("CytoscapeAttributesType.txt", "CytoscapeAttributesTarget.txt"), which can be imported into cytoscape to visualize the network.

Note

The network-table generated with this function can be also visualized in R using the igraph package. The network-table can be transformed into an igraph object using the function "graph.data.frame( )" from igraph.

Examples

Shortest-path subnetwork from *Foxo1* (84482), *Ldha* (24533) to alpha D-glucose ("cpd:C00267") and lactate ("cpd:C00186"). Different source_gene formats are valid:

1) Source_genes as network IDs (in this case orthology IDs): fastest option. To get gene KEGG IDs use "MS_GetKEGG_GeneID( )" as shown below:

```
MS_GetKEGG_GeneID(c("foxo1","ldha"), organism_code = "rno", organism_name = "rat")

subnet_KEGG <- MetaboSignal_NetworkCytoscape(MetaboSignal_table, organism_code =
   "rno", source_genes = c("K07201", "K00016"), target_metabolites = c("cpd:C00267",
      "cpd:C00186"))
```

2) Source_genes as entrez IDs

```
subnet_Entrez <- MetaboSignal_NetworkCytoscape(MetaboSignal_table, organism_code =
   "rno", source_genes = c("84482", "24533"), target_metabolites = c("cpd:C00267",
      "cpd:C00186"))
```

3) Source_genes as symbols

```
subnet_Symbol <- MetaboSignal_NetworkCytoscape(MetaboSignal_table,
   organism_code="rno", organism_name ="rat", source_genes = c("foxo1", "ldha"),
      target_metabolites = c("cpd:C00267", "cpd:C00186"))
```

MetaboSignal support functions

MS_FindKEGG

Description

This function returns a list of entries corresponding to one of the following KEGG databases: "compound", "organism", "pathway". It can also find entries with matching query keywords in a given database.

Usage

MS_FindKEGG(KEGG_database, match = NULL, organism_code = NULL)

Arguments

KEGG_database: character vector containing the name of the KEGG database of interest: "compound", "organism", "pathway".

match: character vector containing one or more elements (i.e. key words) to be matched as compound names.

organism_code: character vector containing the KEGG code for the organism of interest. For example the KEGG code for the rat is "rno". This argument is only required for KEGG_database = "pathway".

Value

By default, a matrix where each row contains the KEGG entries of the database of interest When using the option "match" a list is returned, each list element containing information of matched entries.

Examples

```
MS_FindKEGG(KEGG_database = "compound", match = "acetoacetic acid")

MS_FindKEGG(KEGG_database = "organism", match = c("rattus","human"))

MS_FindKEGG(KEGG_database = "pathway", match = c("glycol", "insulin signal", "akt"),
   organism_code = "rno")
```

MS_FindMappedNodes

Description

This function can be used to find out if a set of genes or metabolites of interest can be mapped onto the MetaboSignal network.

Usage

MS_FindMappedNodes(nodes, network_table, organism_name, orthology = TRUE)

Arguments

nodes: character vector containing the IDs of the genes or the metabolites to be mapped onto the network. All IDs need to correspond to the same molecular entity (i.e. gene or metabolite). For metabolites, KEGG IDs are required (see function "MS_FindKEGG( )"). For genes, entrez IDs or official symbols can be used, but note that all genes need to be in the same ID format (i.e. entrez or symbols). It is preferable to use entrez IDs rather than gene symbols, since some gene symbols are not unique.

network_table: two-column matrix where each row represents and edge between two nodes. See function "MetaboSignal_matrix ( )".

organism_code: character vector containing the KEGG code for the organism of interest. For example the KEGG code for the rat is "rno". This argument is only required for KEGG_database = "pathway".

organism_name: character vector containing the common name of the organism of interest (e.g. "rat", "mouse", "human", "zebrafish") or taxonomy id. For more details, check: http://docs.mygene.info/en/latest/doc/data.html#species. This argument is not required to map metabolites.

orthology: logical scalar indicating whether the gene nodes of the network_table represent KEGG orthology IDs (FALSE) or specific KEGG gene IDs (TRUE). By default, orthology = TRUE.

Value

A list reporting which genes or metabolites can or cannot be mapped onto the network.

Examples

```
# Map entrez IDs onto the network
MS_FindMappedNodes(nodes = c("303565", "24267", "11114"), MetaboSignal_table,
   organism_code = "rno", organism_name = "rat", orthology = TRUE)

# Map gene symbols onto the network
MS_FindMappedNodes(nodes = c("G6pc3","Comt"), MetaboSignal_table, organism_code =
   "rno", organism_name ="rat", orthology = TRUE)

# Map D-glucose ("cpd:C00031") and taurine ("cpd:C00245") onto the network
MS_FindMappedNodes(nodes = c("cpd:C00031","cpd:C00245"), MetaboSignal_table,
   orthology = TRUE)
```

##### MS_GetKEGG_GeneID ####

Description

This function allows transforming entrez gene IDs or official gene symbols into KEGG IDs (orthology IDs or organism-specific gene IDs). The transformed KEGG IDs can be stored and used as source_genes in the functions "MetaboSignal_distances( )" or

"MetaboSignal_NetworkCytoscape( )". This strategy allows reducing the time required to compute these functions.

Usage

MS_GetKEGG_GeneID(genes, organism_code, organism_name, output = "vector", orthology = TRUE)

Arguments

genes: character vector containing the entrez ID or official symbols of the genes of interest. All genes need to be in the same ID format (i.e. entrez or symbols). It is preferable to use entrez IDs rather than gene symbols, since some gene symbols are not unique.

organism_code: character vector containing the KEGG code for the organism of interest. For example the KEGG code for the rat is "rno". This argument is only required for KEGG_database = "pathway".

organism_name: character vector containing the common name of the organism of interest (e.g. "rat", "mouse", "human", "zebrafish") or taxonomy id. For more details, check: http://docs.mygene.info/en/latest/doc/data.html#species. This argument is only required when gene symbols are used.

output: character constant indicating whether the function will return a vector containing mapped and transformed KEGG IDs (output = "vector"), or a matrix containing both mapped entrez IDs or gene symbols and their corresponding KEGG IDs (output = "matrix"). By default, output = "vector".

orthology: logical scalar indicating whether the genes IDs will be transformed into orthology IDs or into organism-specific KEGG gene IDs. By default, orthology = TRUE.

Value

A character vector containing mapped and transformed KEGG IDs or a matrix containing both mapped entrez IDs or gene symbols and their corresponding KEGG IDs.

Examples

Transform gene symbol *Hoga1* (293949) into rat-specific KEGG ID

MS_GetKEGG_GeneID(genes = "Hoga1", organism_code = "rno", organism_name = "rat", orthology = FALSE)

MS_GetKEGG_GeneID(genes = "Hoga1", "rno", "rat", output = "matrix", orthology = FALSE)

Transform entrez ID 293949 into orthology KEGG ID

MS_GetKEGG_GeneID(genes = "293949", organism_code = "rno", output = "matrix")

MS_ChangeNames

Description

This function allows transforming KEGG IDs of genes or metabolites into their corresponding common names (for metabolites) or symbols (for genes).

Usage

MS_ChangeNames(nodes, organism_code)

Arguments

nodes: character vector or matrix containing the KEEG IDs of either metabolites, genes (organism-specific or orthology), or reactions.

organism_code: character vector containing the KEGG code for the organism of interest. For example the KEGG code for the rat is "rno". See the function "MS_FindKEGG( )". This argument is ignored when nodes are metabolites.

Value

A character string or a matrix containing the common metabolite names or gene symbols corresponding to the input KEGG IDs. Reaction IDs remain unchanged.

Examples

MS_ChangeNames(c("rno:84482", "K01084", "cpd:C00267"), "rno")

MS_GetShortestpaths

Description

This function calculates the shortest path(s) between any two reachable nodes of a network-table.

Usage

MS_GetShortestpaths(network_table, source_node, target_node, mode = "SP", type = "first")

Arguments

network_table: two-column matrix where each row represents and edge between two nodes. See function "MetaboSignal_matrix ( )".

source_node: character vector containing the ID of the node from which the shortest paths will be calculated.

target_node: character vector containing the ID of node to which the shortest path will be calculated.

mode: character constant indicating whether a directed or an undirected network will be considered. "all" indicates that all the edges of the network will be considered as undirected. "out" indicates that all the edges of the network will be considered as directed. "SP" indicates that all network will be considered as directed except the edges linked to target metabolite,

which will be considered as undirected. The difference between the "out" and "SP" options, is that the latter aids reaching target metabolites that are substrates of irreversible reactions. By default, mode = "SP".

type: character constant indicating whether all shortest paths or a single shortest path will be considered when there are several shortest paths between a source_gene and a target_metabolite. If type = "all", all shortest paths will be considered. If type = "first" a single path will be considered. If type = "bw" the path with the highest betweenness score will be considered. The betweenness score is calculated as the average betweenness of the gene nodes of the path. Note that using type = "bw" increases the time required to compute this function. By default, type = "first".

Value

A vector or a matrix where each row contains a shortest path from the source_node to the target_node. KEGG IDs can be transformed into common names using the function "MS_ChangeNames( )".

Examples

```
# All shortest paths from G6PC ("K01084") to pyruvate ("cpd:C00022")
path1 <- MS_GetShortestpaths(MetaboSignal_table, "K01084", "cpd:C00022",  mode = "SP",
   type = "all")

# Bw-ranked shortest path from G6PC ("K01084") to pyruvate ("cpd:C00022")
path2 <- MS_GetShortestpaths(MetaboSignal_table, "K01084", "cpd:C00022",  mode = "SP",
   type = "bw")

# Shortest path from HK ("K00844") to alpha-D-Glucose ("cpd:C00267") (substrate of irreversible reaction) with mode = "SP"
path3 <- MS_GetShortestpaths(MetaboSignal_table, "K00844", "cpd:C00267", mode = "SP",
   type = "first")

# Shortest path from HK ("K00844") to alpha-D-Glucose ("cpd:C00267") (substrate of irreversible reaction) with mode = "out"
path4 <- MS_GetShortestpaths(MetaboSignal_table, "K00844", "cpd:C00267", mode = "out",
   type = "first")
```

##### MS_NodeBW ####

Description

This function calculates the betweenness of each node of the network.

Usage

MS_NodeBW(network_table, mode = "all", normalized = TRUE)

Arguments
network_table: two-column matrix where each row represents and edge between two nodes. See function "MetaboSignal_matrix ( )".

mode: character constant indicating whether a directed ("out") or undirected ("all") network will be considered. By default, mode = "all".

normalized: logical scalar indicating whether to normalize the betweenness scores. If TRUE, normalized betweenness scores will be returned. If FALSE, raw betweenness scores will be returned. By default, normalized = TRUE.

Value
A numeric vector containing the betweenness of each node of the network. The function also produces and histogram showing the distribution of node betweenness.

Examples
MS_NodeBW(MetaboSignal_table)

##### MS_FilterNetwork ####

Description
This function allows reducing the dimensionality of a network, by removing nodes that do not meet the established distance and/or node betweenness criteria.

Usage
MS_FilterNetwork(network_table, mode = "all", type, target_node, distance_th, bw_th)

Arguments
network_table: two-column matrix where each row represents and edge between two nodes. See function "MetaboSignal_matrix ( )".

mode: character constant indicating whether a directed ("out") or undirected ("all") network will be considered. By default, mode = "all".

type: character constant used to establish the criteria for filtering the network. "bw" indicates that edges (i.e. rows of the network_table) containing at least one node with betweenness below bw_th will be neglected. "distance" indicates edges containing at least one node with shortest path length to the target_node above distance_th will be neglected. "all" indicates that edges containing at least one node with either betweenness below bw_th or distance above distance_th, will be neglected.

target_node: character vector containing the ID of the node to which the distances will be calculated.

distance_th: numeric value corresponding to the distance threshold. Nodes with shortest path length to the target_node above this threshold will be removed from the network-table.

bw_th: numeric value corresponding to the normalized-betweenness threshold. Nodes with betweenness below this threshold will be removed from the network-table. See also "MS_NodeBW( )".

Value

A two-column matrix where each row represents an edge between two nodes.

Examples

Remove edges containing nodes with distance to D-glucose ("cpd:C00031") > 2

```
network_filtered1 <- MS_FilterNetwork(MetaboSignal_table, type = "distance", target_node =
   "cpd:C00031", distance_th = 2)
```

Remove edges containing nodes with distance to D-glucose ("cpd:C00031") > 2 or normalized-betweenness < 0.00005

```
network_filtered2 <- MS_FilterNetwork(MetaboSignal_table, type = "all", target_node =
   "cpd:C00031", distance_th = 2, bw_th = 0.00005)
```

Note below that network_filtered1 has one edge more than network_filtered2. This is because "cpd:C00031" has betweenness = 0, and therefore it is removed in network_filtered2:

```
setdiff(as.vector(network_filtered1),as.vector(network_filtered2))
```

##### MS_RemoveNode ####

Description

This function allows removing undesired nodes of the network-table.

Usage

MS_RemoveNode(nodes, network_table)

Arguments

nodes: character vector containing the node IDs to be removed.

network_table: two-column matrix where each row represents and edge between two nodes. See function "MetaboSignal_matrix ( )".

Value

A two-column matrix corresponding to the input network-table without the undesired nodes.

Examples

Remove glucose nodes

```
MetaboSignal_table_GlucoseRemoved <- MS_RemoveNode(nodes = c("cpd:C00267",
   "cpd:C00221",  "cpd:C00031"), MetaboSignal_table)
```

MS_ReplaceNode

Description

This function allows replacing node IDs of a network-table. It can be used to cluster the IDs of chemical isomers (e.g. alpha-D-glucose ("cpd:C00267"), D-glucose ("cpd:C00031"), and beta-D-glucose ("cpd:C00021")) into a single ID.

Usage

MS_ReplaceNode(node1, node2, network_table)

Arguments

node1: character vector containing the node IDs to be replaced.

node2: character vector containing the ID that will be used as a replacement.

network_table: two-column matrix where each row represents and edge between two nodes. See function "MetaboSignal_matrix ( )".

Value

A two-column matrix corresponding to the input network-table with replaced nodes.

Examples

Cluster D-glucose isomers ("cpd:C00267","cpd:C00221","cpd:C00031") into a common node ("cpd:C00031")

```
MetaboSignal_table_GlucoseClustered <- MS_ReplaceNode(node1 = c("cpd:C00267",
   "cpd:C00221"), node2 = "cpd:C00031", MetaboSignal_table)
```

MS_ToCytoscape

Description

The function exports a network file ("CytoscapeNetwork.txt") and two attribute files ("CytoscapeAttributesType.txt", "CytoscapeAttributesTarget.txt"), which can be imported into cytoscape to visualize the network. The first attribute file allows customizing the nodes of the network based on the molecular entity they represent: metabolites, metabolic-genes, or signaling-genes. The second attribute file allows discriminating a set of nodes of interest ("target") from any other node ("untarget") of the network.

Usage

MS_ToCytoscape (network_table, organism_code, names = TRUE, target_nodes = NULL, file_name = "Cytoscape")

Arguments

network_table: two-column matrix where each row represents and edge between two nodes. See function "MetaboSignal_matrix ( )".

organism_code: character vector containing the KEGG code for the organism of interest. For example the KEGG code for the rat is "rno". See the function "MS_FindKEGG( )".

names: logical scalar indicating whether the metabolite or gene KEGG IDs will be transformed into common metabolite names or gene symbols. Reaction IDs remain unchanged. By default, names = TRUE.

target_nodes: character vector containing the IDs of the target nodes to be discriminated from the other nodes of the network.This argument is optional.

file_name: character vector that allows customizing the name of the exported files. By default, the file_name = "Cytoscape".

Value

A data frame where each row represents an edge between two nodes. The function also generates and exports a network file ("CytoscapeNetwork.txt") and two attribute files ("CytoscapeAttributesType.txt", "CytoscapeAttributesTarget.txt"), which can be imported into cytoscape to visualize the network.

Examples

```
# Export glycolysis to cytoscape
Glucolysis <- MetaboSignal_matrix(metabo_paths = "mmu00010", organism_name ="mouse")
MS_ToCytoscape(Glucolysis, organism_code = "mmu")
```

MetaboSignal workflow

In order to illustrate the functionality of our package, we have used transcriptomic and metabonomic datasets from white adipose tissue of rat congenic strains derived from the diabetic Goto-Kakizaki (GK) and normoglycemic Brown-Norway (BN) rats.

We next describe how MetaboSignal was used to provide a mechanistic explanation of the gene-metabolite associations found in this study. As an example, we will use the associations between the genes: *G6pc3* (303565), *Ship2* (65038) or *Ppp2r5b* (309179) with D-glucose.

1) Define input data

We built a rat-specific MetaboSignal network by merging two metabolic pathways: "glycolysis" and "inositol phosphate metabolism", with two signaling pathways: "insulin signaling pathway" and "PI3K-Akt signaling pathway".

We used the function "MS_FindKEGG( )" to find the "organism_code" of the rat and the IDs of the pathways of interest.

```
MS_FindKEGG(KEGG_database = "organism", match = "rattus")

## $rattus
##                                         T
##                                "T01003"
##                           organism_code
##                                   "rno"
##                           organism_name
##                "Rattus norvegicus (rat)"
##                             description
## "Eukaryotes;Animals;Vertebrates;Mammals"

MS_FindKEGG(KEGG_database = "pathway", match = c("glycol", "inositol phosphate",
    "insulin signal", "akt"), organism_code = "rno")

## $glycol
##                                                        path_ID
##                                              "path:rno00010"
##                                             path_Description
## "Glycolysis / Gluconeogenesis - Rattus norvegicus (rat)"
##
## $`inositol phosphate`
##                                                         path_ID
##                                               "path:rno00562"
##                                              path_Description
## "Inositol phosphate metabolism - Rattus norvegicus (rat)"
##
## $`insulin signal`
##                                                     path_ID
##                                           "path:rno04910"
##                                          path_Description
## "Insulin signaling pathway - Rattus norvegicus (rat)"
##
## $akt
##                                                      path_ID
##                                            "path:rno04151"
##                                           path_Description
## "PI3K-Akt signaling pathway - Rattus norvegicus (rat)"
```

Based on this,

```
metabo_paths <- c("rno00010", "rno00562")
signaling_paths <- c("rno04910", "rno04151")
```

2) Build MetaboSignal network-table

We used the selected metabo_paths and signaling_paths to build a MetaboSignal network-table. Since we focused on an adipose tissue dataset, we decided to exclude signaling genes not expressed in soft tissue.

```
# Adipose tissue-filtered network
MetaboSignal_table <- MetaboSignal_matrix(metabo_paths = metabo_paths,
    signaling_paths = signaling_paths, organism_name = "rat",
        tissue = c("soft tissue 1", "soft tissue 2"))

# Unfiltered-network
MetaboSignal_tableUnfiltered <- MetaboSignal_matrix(metabo_paths = metabo_paths,
    signaling_paths = signaling_paths)

# Check signaling-genes removed by tissue-filtering
neglected_genes <- MS_ChangeNames(setdiff(as.vector(MetaboSignal_tableUnfiltered),
    as.vector(MetaboSignal_table)), "rno")
```

MetaboSignal_table is a two-column matrix where each row represents an edge between two nodes (from the node in column 1 to node in column 2). Since we did not use the option "expand_genes", the gene nodes of the network represent orthology KEGG IDs. Note that clustering gene-isoforms by orthology IDs is a very convenient strategy to reduce network dimensionality without loosing biological information.

In total, 24 signaling genes were excluded from the network for not being expressed in adipocytes. Examples of these genes are: *Kit*, *Fgfr3*, *Elk1*, *Csf3r*, *Il4r*, *Nos3*.

3) Customize MetaboSignal network-table

Given that we were not interest in discriminating between different isomers of D-glucose, we used the function "MS_ReplaceNodes( )" to group the IDs of alpha-D-glucose ("cpd:C00267"), beta-D-glucose ("cpd:C00221") and D-glucose ("cpd:C00031").

```
MetaboSignal_table <- MS_ReplaceNode(node1 = c("cpd:C00267", "cpd:C00221"),
    node2 = "cpd:C00031", MetaboSignal_table)
```

We used the function "MS_FindMappedNodes( )" to check that the glucose isomers had been successfully clustered.

```
MS_FindMappedNodes(nodes = c("cpd:C00267", "cpd:C00221", "cpd:C00031"),
    MetaboSignal_table)

## $`metabolites mapped onto the network`
## [1] "cpd:C00031"
##
## $`metabolites not mapped onto the network`
## [1] "cpd:C00267" "cpd:C00221"
```

4) Build distance matrix

We used the function “MetaboSignal_distances( )” to calculate the shortest path lengths from our genes of interest: *G6pc3* (303565), *Ship2* (65038) or *Ppp2r5b* (309179) to D-glucose ("cpd:C00031"). We used the default mode (mode = "SP"), which indicates that all the network is considered as directed, except the edges linked to the target metabolite (in this case D-glucose) that are considered as undirected. This option is designed to reach metabolites acting as substrates of irreversible reactions, while keeping the directionality of the network.

```
MetaboSignal_distances(MetaboSignal_table, organism_code = "rno", source_genes = c
    ("303565", "65038", "309179"), target_metabolites = "cpd:C00031", names = TRUE)

##        D-Glucose
## G6PC           1
## SHIP2          4
## PPP2R5         3
```

5) Build shortest-paths subnetwork

Finally, we used the function “MetaboSignal_NetworkCytoscape( )” to build a subnetwork containing betweenness-ranked shortest paths from our genes of interest: *G6pc3* (303565), *Ship2* (65038) or *Ppp2r5b* (309179) to D-glucose ("cpd:C00031").

```
subnetwork <- MetaboSignal_NetworkCytoscape(MetaboSignal_table, organism_code =
    "rno", source_genes = c("303565", "65038", "309179"), target_metabolites =
        "cpd:C00031", type = "bw", file_name = "MSCytoscape")
```

This function exported three files in the working directory: "MSCytoscapeNetwork.txt", "MSCytoscapeAttributesType.txt", and "MSCytoscapeAttributesTarget.txt". These files were imported into cytoscape (see guidelines) to build the following network:

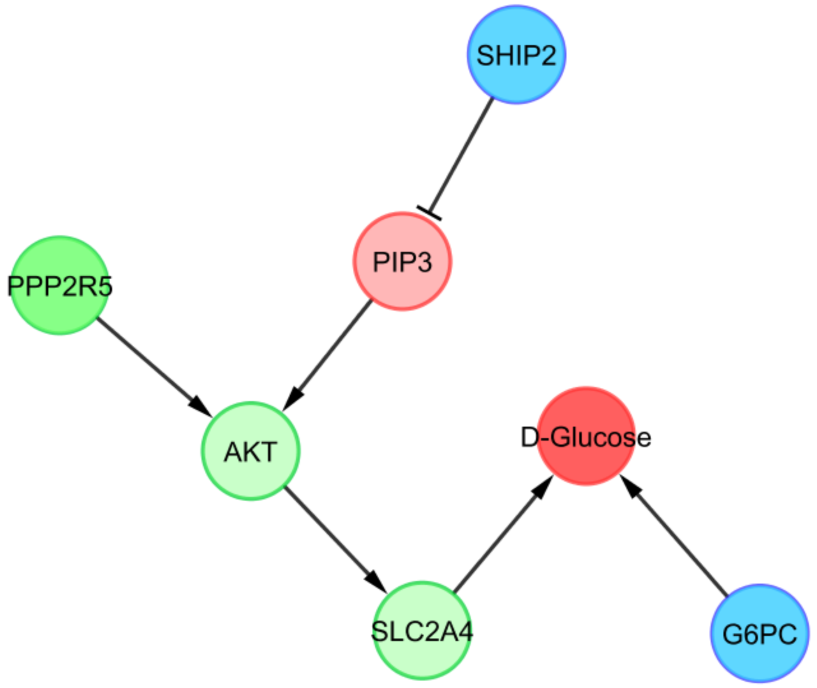

Cytoscape (2.8.2) guidelines

Import network file

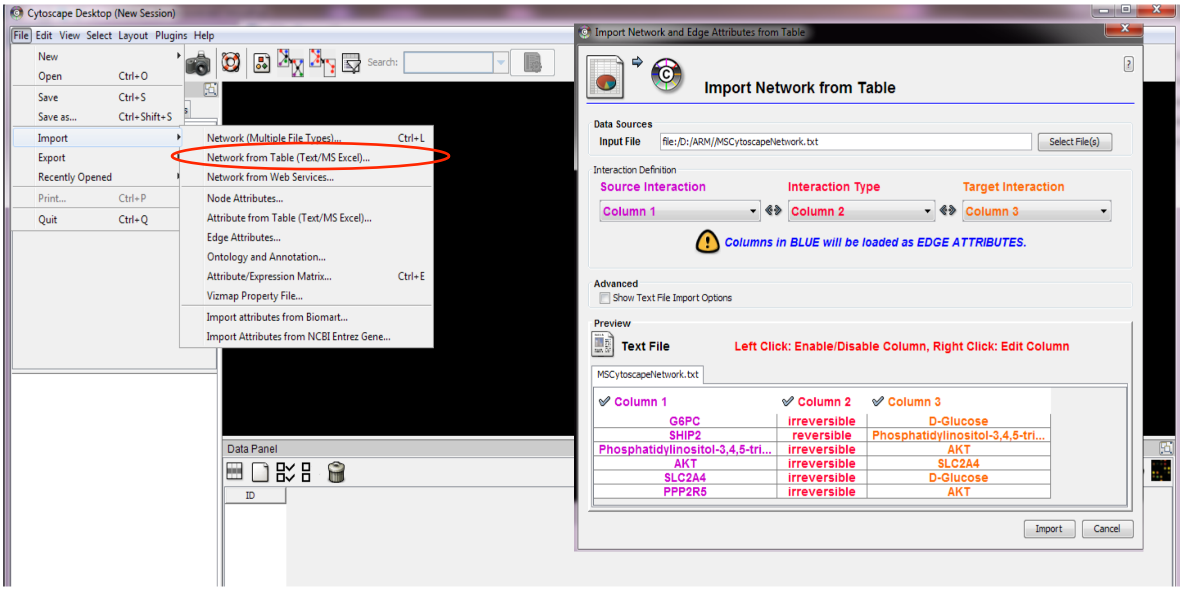

Import attribute files

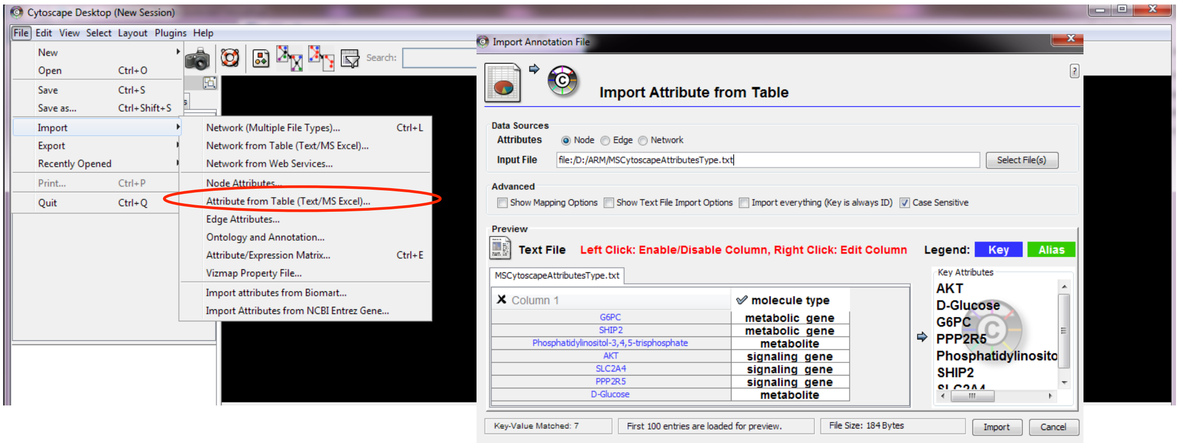

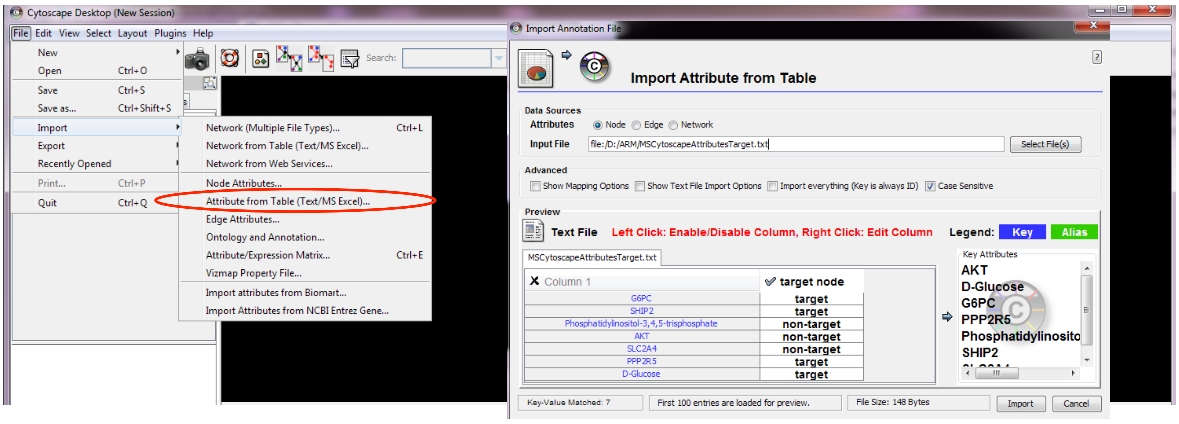

Customize network based on attribute files using VizMapper

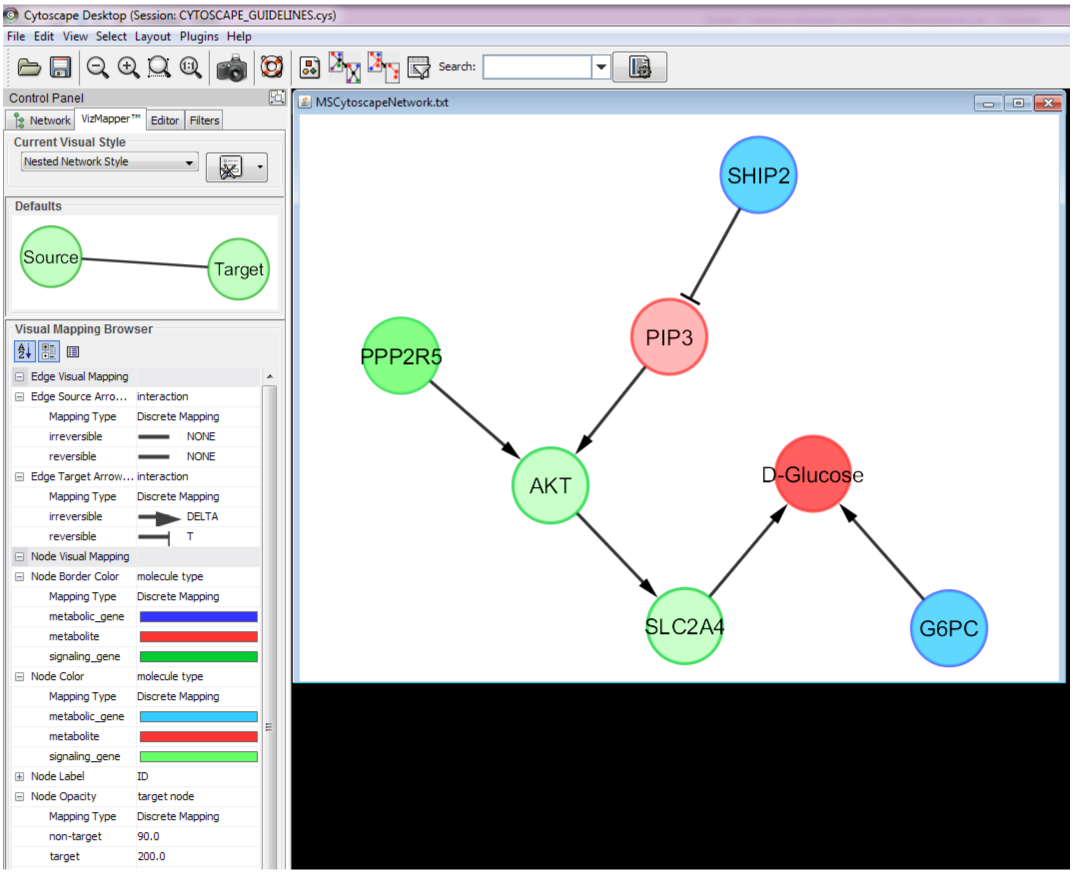

Export network

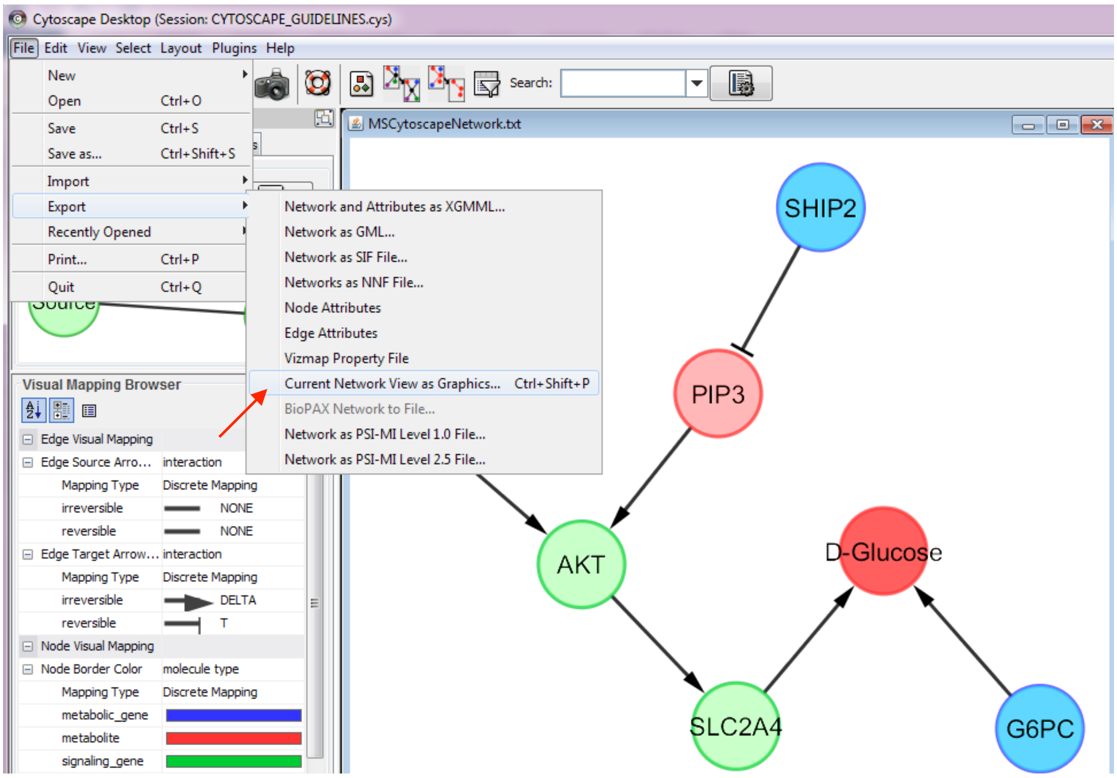

Supplement: Supplementary Data [file btw697_supp.pdf]
